# CXC chemokine receptor 1 predicts postoperative prognosis and chemotherapeutic benefits for TNM II and III resectable gastric cancer patients

**DOI:** 10.18632/oncotarget.12815

**Published:** 2016-10-21

**Authors:** Yifan Cao, Hao Liu, Heng Zhang, Chao Lin, Ruochen Li, Songyang Wu, He Li, Hongyong He, Weijuan Zhang, Jiejie Xu

**Affiliations:** ^1^ Department of Biochemistry and Molecular Biology, School of Basic Medical Sciences, Fudan University, Shanghai, China; ^2^ Department of General Surgery, Zhongshan Hospital, Fudan University, Shanghai, China; ^3^ Department of Immunology, School of Basic Medical Sciences, Fudan University, Shanghai, China

**Keywords:** CXCR1, gastric cancer, nomogram, overall survival, adjuvant chemotherapy

## Abstract

**Backround:**

Abnormal expression of CXC chemokine receptor 1 (CXCR1) has shown the ability to promote tumor angiogensis, invasion and metastasis in several cancers. The purpose of our curret study is to discover the clinical prognostic significance of CXCR1 in resectable gastric cancer.

**Methods:**

330 gastric cancer patients who underwent R0 gastrectomy with standard D2 lymphadenectomy at Zhongshan Hospital, Fudan University between 2007 and 2008 were enrolled. CXCR1 expression was evaluated with use of immunohistochemical staining. The relation between CXCR1 expression and clinicopathological features and postoperative prognosis was respectively inspected.

**Results:**

In both discovery and validation data sets, CXCR1 high expression indicated poorer overall survival (OS) in TNM II and III patients. Furthermore, multivariate analysis identified CXCR1 expression and TNM stage as two independent prognostic factors for OS. Incorporating CXCR1 expression into current TNM staging system could generate a novel clinical predictive model for gastric cancer, showing better prognostic accuracy with respect to patients’ OS. More importantly, TNM II patients with higher CXCR1 expression were shown to significantly benefit from postoperative 5-fluorouracil (5-FU) based adjuvant chemotherapy (ACT).

**Conclusion:**

CXCR1 in gastric cancer was identified as an independent adverse prognostic factor. Combining CXCR1 expression with current TNM staging system could lead to better risk stratification and more accurate prognosis for gastric cancer patients. High expression of CXCR1 identified a subgroup of TNM stage II gastric cancer patients who appeared to benefit from 5-FU based ACT.

## INTRODUCTION

Although the majority of European and Northern American countries have seen a steady decline in the incidence and mortality rates of gastric cancer since the middle of the 20^th^ century, gastric cancer is still common and acts as a leading cause of cancer death within a number of less developed countries [1]. In China, the latest cancer statistics show that gastric cancer is the second most common cancer diagnosed all over the country [2]. An increasing amount of evidence has emerged that gastric cancer represents a number of cancers correlated with inflammation [3]. Though the detailed mechanisms underlying tumorigenesis in gastric cancer still remain unclear, chronic gastritis derived from *Helicobacter pylori* infection is considered as a major risk factor for human gastric cancer [4]. Surgical resection is believed to be the only feasible curative treatment for gastric cancer [5]. Nevertheless, many gastric cancer patients are diagnosed at late stage due to the atypical symptoms which they neglected in the early stage of the disease. As a result, high rate of relapse in those patients emphasizes the significance to take adjuvant therapy into consideration. As to patients with late stage gastric cancer, 5-fluorouracil (5-FU) based adjuvant chemotherapy (ACT) is generally applied as first-line postoperative treatment [6]. However, whether to use adjuvant therapy in gastric cancer is still controversial because the survival in many randomized studies still lacks significant benefit from adjuvant therapy [7]. Therefore, there is an urgent need for a novel gastric cancer classification that can be applied for more precise prediction of patient outcomes and treatment response. Existing prognostic model for gastric cancer risk stratification and treatment strategy is predominantly established on the basis of tumor cell-oriented stratification systems, such as TNM stage. However, the prognostic power of such stratification system is limited, due to the neglect of the information derived from tumor microenvironment. Consequently, the combination of tumor-microenvironment information with TNM staging system might improve the prognostic accuracy to a large extent.

Since gastric cancer is a kind of inflammation-associated cancer, increasing evidence has confirmed that such inflammatory cytokines as CXC chemokines, are correlated with tumor progression and host anti-tumor response [8, 9]. According to the absence or the presence of a Glu-Leu-Arg composed ELR motif, CXC chemokines are respectively divided into ELR-CXC chemokines and ELR+CXC chemokines. The latter, including Interleukin-8 (IL-8) [10–12], can play a key role in inflammatory responses and promote angiogenesis and cell proliferation [9]. It was shown that IL-8 could be up-regulated in several cancers and resulted in poor prognosis [13, 14]. Especially, IL-8 was also found overexpressed in gastric mucosa infected with *Helicobacter pylori (H. pylori)* [15], a major etiological factor for gastric cancer [4]. More importantly, the expression of IL-8 could directly lead to a poor prognosis in gastric cancer [15]

CXC chemokine receptor 1 (CXCR1) is a class-A, rhodopsin-like G-protein-coupled receptor (GPCR), which takes charge of cellular signal transduction and can be also targeted as a drug receptor [16]. CXCR1 functions as a high-affinity receptor for IL-8, and IL-8 is a major mediator of inflammatory responses and tumorigenesis as mentioned above [17–19]. CXCR1 has proved to be correlated with a number of cancers, including breast cancer [20, 21], prostate cancer [22], colorectal cancer [23] and lung cancer [24]. Nevertheless, the exact role which CXCR1 plays in gastric cancer still remains unclear and needs further investigation.

In our current study, we planned to discover the clinical prognostic effect of CXCR1 on resectable gastric cancer patients. The expression of CXCR1 in human gastric cancer tissues was evaluated by means of immunohistochemistry. The relation between CXCR1 expression and clinical outcomes was inspected as well. These results may not only shed light on the clinical significance of CXCR1 in gastric cancer, but pave the way to a promising prognostic system which can evaluate the outcomes for gastric cancer patients and identify those who are recommended to receive ACT as well.

## PATIENTS AND METHODS

### Patients and tissue samples

330 consecutive gastric cancer patients who underwent R0 gastrectomy with standard D2 lymphadenectomy between August 2007 and December 2008 at Zhongshan Hospital, Fudan University (Shanghai, China) were recruited in our study. The total 330 patients were randomly-assigned into two independent patient cohorts: discovery data set (*n* = 158) and validation data set (*n* = 172). Clinicopathological characteristics of these patients, including age, gender, tumor localization, tumor size, differentiation, Lauren classification, T classification, N classification, TNM stage and the use of adjuvant chemotherapy, were retrospectively collected. All specimens were obtained from the patients who had been informed of the consent approved by the Clinical Research Ethics Committee of Zhongshan Hospital. The TNM staging system was performed according to the 2010 International Union Against Cancer TNM classification system. The specimens were all evaluated independently by two gastroenterology pathologists who were blind to patients’ clinicopathological data. The endpoint of interest was overall survival (OS). With respect to patients, OS were computed from the date of receiving gastrectomy to the date of death or the last follow-up. Patients were observed until April 2014. For discovery data set, the range of follow-up time was from 2 months to 76 months, and the median follow-up time was 52 months. As to validation data set, the follow-up time ranged from 2 months to 79 months and the median follow-up time was 41months.

### Tissue microarray and immunohistochemistry

After targeting optimal tumor content on hematoxylin and eosin-stained slides, we sought to construct tumor tissue microarray (TMA) slides. The tissue microarrays were processed as described elsewhere [25]. In brief, two tissue cores for one patient were extracted from each representative tumor tissue and the gastric tissue adjacent to the tumor within 5cm to manufacture the TMA slides. Immunohistochemistry for CXCR1 was carried out according to avidin-biotin complex method (ABC; Vector Laboratories, Burlingame, CA). Incubation with monoclonal antibody against CXCR1 (1:800 dilution, R&D Systems, Minneapolis, MN) was performed at 4°C for 18 hours [26]. To guarantee an objective comparison between different samples, the time and temperature were strictly controlled for every single tissue microarray during the immunohistochemistry. The whole set of tissue specimens was also processed and immunostained at the same time as well.

### Evaluation of immunostaining intensity

The immunostaining intensity of CXCR1 was evaluated by two independent observers who were specialized in IHC staining intensity assessment. Both of the observers were blind to patients’ clinicopathological data. The IHC staining intensity assessment was performed as previously described [27]. In brief, the IHC staining intensity was composed of staining degree and staining extent. The staining degree was stratified as 0 (negative staining), 1 (weak staining), 2 (moderate staining) and 3 (strong staining), while the staining extent was defined as the percentage of positive cancer cells (0-100%). The IHC staining degree and extent were multiplied to generate a CXCR1 IHC staining intensity score ranging from 0 to 300. All tissue microarrays were scored independently by two gastroenterology pathologists and paired at the end. Especially, if the two scores given by two independent observers were discordant for a certain tumor tissue and the variability of difference was more than 5%, then the discordant case would be reviewed to reach the final consensus score, or an average value of the two discordant scores was chosen. The cut-off value for the definition of high/low CXCR1 expression subgroups was the median value. The correlation between CXCR1 expression and survival outcomes was inspected by a third investigator who did not participate in the scoring process.

### Statistical analysis

Statistical analysis was performed *via* SPSS Statistics 21.0 (SPSS Inc., Chicago, IL); Pearson's chi-squared test or Fisher's exact test was applied for categorical variables and continuous variables were analyzed by means of *t* test. Survival curves were constructed through the Kaplan-Meier method and the significance of the difference between survival curves was assessed with the log-rank test. The Cox proportional hazards regression model was utilized for multivariate analysis. The nomogram analysis and calibration curve were established with use of the R software version 3.0.2 and the ‘rms’ package (R Foundation for Statistical Computing, Vienna, Austria). Harrell's index of concordance (C-index) and Akaike information criterion (AIC) were respectively calculated to compare the accuracy of different predictive models. All tests were two-sided, and *P* < 0.05 was considered to be statistically significant.

## RESULTS

### Immunohistochemical intensity of CXCR1 and its correlation with pathological characteristics

**Figure 1:**
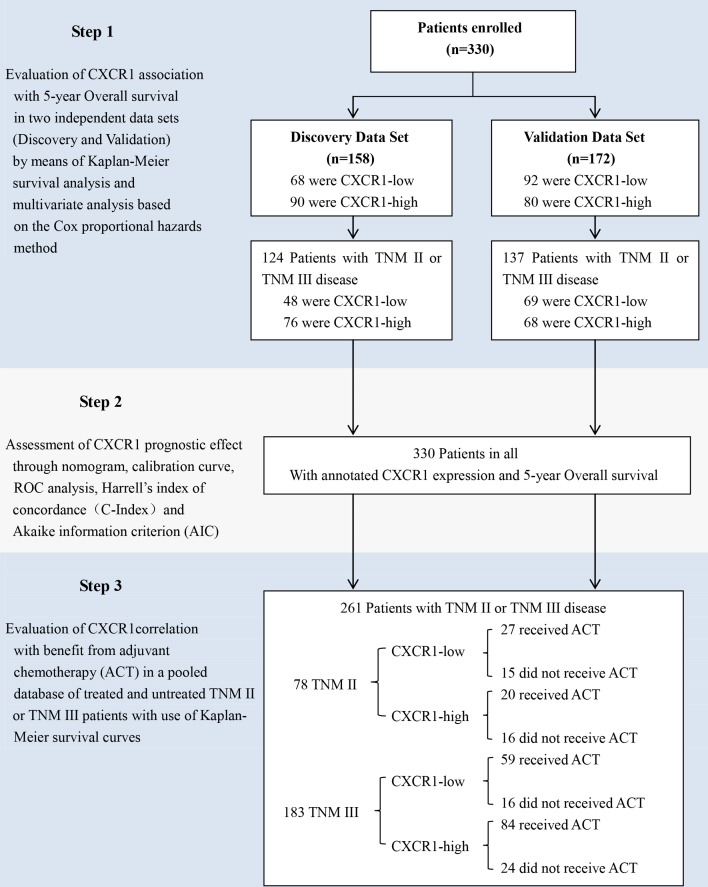
Study design A total of 330 consecutive patients who suffered from resectable gastric cancer underwent R0 gastrectomy with standard D2 lymphadenectomy between August 2007 and December 2008 in Zhongshan Hospital, Fudan University (Shanghai, China) was enrolled in the study. The relation between CXCR1 expression and overall survival was tested in two independent randomly-assigned patient cohorts: discovery data set and validation data set. The association between CXCR1 expression and benefit from adjuvant chemotherapy (ACT) was tested in a pooled database of 78 patients with TNM stage II disease and 183 patients with stage III disease from the two independent data sets.

**Figure 2:**
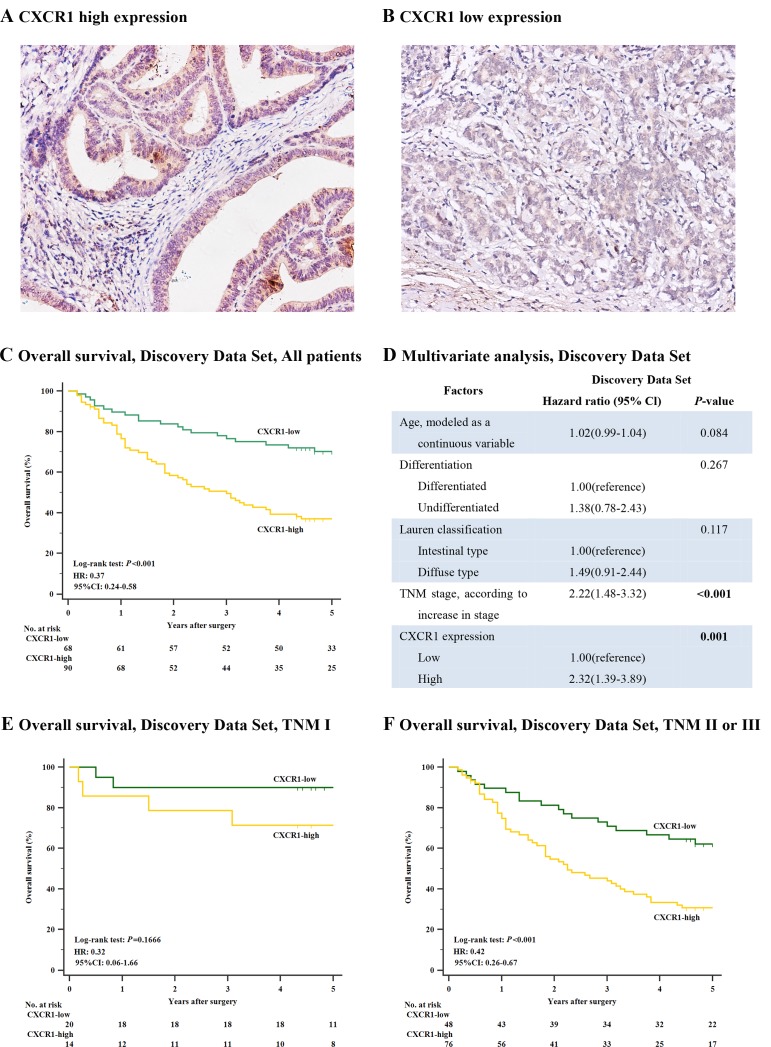
Correlation between CXCR1 expression and overall survival in the discovery data set Kaplan-Meier analysis of overall survival was performed according to CXCR1 expression in patients with resectable gastric cancer. (**Panel A**.) CXCR1 high expression in human gastric cancer sample. Magnification: ×200. (**Panel B**.) CXCR1 low expression in human gastric cancer sample. Magnification: ×200. (**Panel C**.) Overall survival, discovery data set, all patients (*n* = 158, *P* < 0.001, HR: 0.37, 95% CI: 0.24-0.58). (**Panel D**.) Multivariate analysis, discovery data set. (**Panel E**.) Overall survival, discovery data set, TNM I patients (*n* = 34, *P* = 0.1666). (**Panel F**.) Overall survival, discovery data set, TNM II or III patients (*n* = 124, *P* < 0.001, HR: 0.42, 95% CI: 0.26-0.67). *P*-values were calculated by log-rank test.

Ours study was carried out as described in Figure 1. In order to inspect the relation between CXCR1 immunohistochemical intensity and gastric cancer progression, first of all we evaluated CXCR1 expression through IHC staining analysis in the total of 330 gastric cancer patients. Positive staining of CXCR1 was mainly situated on the membrane and/or in the cytoplasm (Figure 2A and 2B). The comprehensive characteristics of patients, together with clinicopathological features, are listed in Table 1.

**Table 1:** Relationship between CXCR1 expression and clinical characteristics

Factors	Discovery Data Set			Validation Data Set		
	CXCR1 expression			CXCR1 expression		
	Low	High	*P*-value	Low	High	*P*-value
All patients	68	90		92	80	
Age(years)^a^ Median (IQR)	61 (53-68)	59 (53-69)	0.629	62(54-69)	57(50-66)	0.984
Gender			0.384			0.969
Female Male	19 49	31 59		29	25	
				63	55	
Localization			0.577			0.951
Proximal Middle Distal	14 11 43	25 14 51		28 10 54	26 8 46	
Tumor size(cm)^a^ Median (IQR)	3 (2-5)	4 (3-5)	0.706	3(2-4)	3.5(2-5)	0.493
Differentiation			0.580			0.481
Differentiated	25	37		37	28	
Undifferentiated	43	53		55	52	
Lauren classification			0.432			0.502
Intestinal type Diffuse type	42	61		62	50	
	26	29		30	30	
T classification			0.081			0.176
T1 T2 T3 T4	15 12 14 27	11 11 14 54		18 12 20 42	7 14 15 44	
N classification			0.107			0.068
N0 N1 N2 N3	33 7 13 15	29 9 17 35		33 12 21 26	19 6 18 37	
TNM stage			**0.014**			0.173
I II III	20 22 26	14 21 55		23 20 49	12 15 53	
Adjuvant chemotherapy^b^			0.545			0.559
No	32	38		35	27	
Yes	36	52		57	53	

Abbreviations: CXCR1= CXC chemokine receptor 1; TNM = tumor node metastasis; *P*-value < 0.05 marked in bold font shows statistical significance.

^a^Modeled as a continuous variable.

^b^Patients with adjuvant chemotherapy received at least one cycle of 5-fluoruracil based chemotherapy.

### Kaplan-Meier survival analysis to evaluate the prognostic capability of CXCR1 in resectable gastric cancer

**Figure 3:**
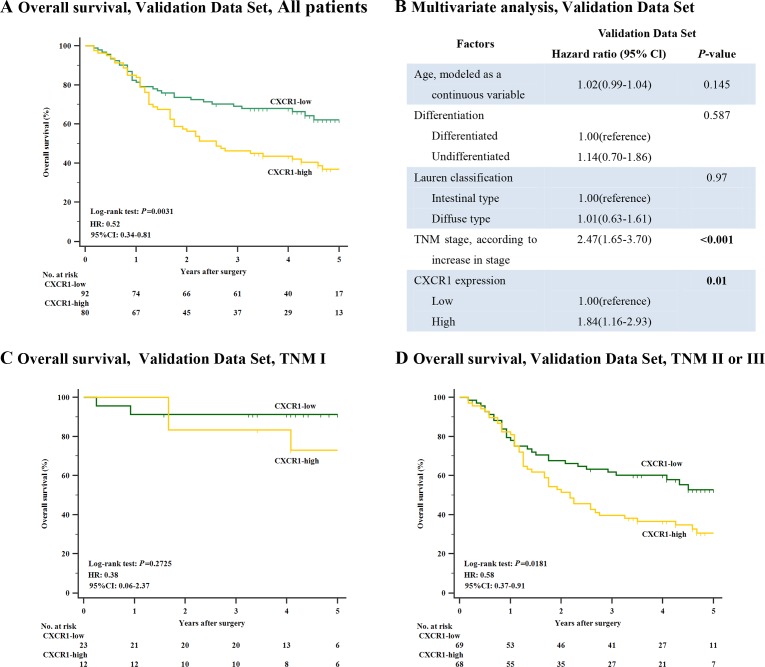
Relation between CXCR1 expression and overall survival in the validation data set (**Panel A**.) Overall survival, validation data set, all patients (*n* = 172, *P* = 0.0031, HR: 0.52, 95% CI: 0.34-0.81). (**Panel B**.) Multivariate analysis, validation data set. (**Panel C**.) Overall survival, validation set, TNM I patients (*n* = 35, *P* = 0.2725). (**Panel D**.) Overall survival, validation set, TNM II or III patients (*n* = 137, *P* = 0.0181, HR: 0.58, 95% CI: 0.37-0.91). *P*-values were calculated by log-rank test.

Next, so as to discover the prognostic capability of CXCR1 in resectable gastric cancer, we used Kaplan-Meier survival analysis to compare OS based on the expression of CXCR1. In both discovery data set and validation data set, patients with low CXCR1 expression had obviously better 5-year OS (*P* < 0.001, Hazard Ratio (HR): 0.37, 95% Confidence Interval (CI): 0.24-0.58 and *P* = 0.0031, HR: 0.52, 95% CI: 0.34-0.81, respectively; Figure 2C and Figure 3A) than those with high CXCR1 expression, implying a vital influence of CXCR1 expression on clinical outcome of resectable gastric cancer patients. Moreover, in order to investigate whether CXCR1 was able to stratify patients of different TNM stage, we consequently classified patients into early-stage (TNM I) disease subgroup and advanced-stage (TNM II or III) disease subgroup. As a result, in TNM I disease, neither discovery data set nor validation data set showed statistical significance in the difference of CXCR1-low and CXCR1-high patients’ overall survival (*P* = 0.1666 and *P* = 0.2725, respectively; Figure 2E and Figure 3C). However, patients with TNM II or III tumors could be obviously stratified by CXCR1 in terms of OS in either discovery data or validation data set (*P* < 0.001 and *P* = 0.0181, respectively; Figure 2F and Figure 3D). Consequently, high expression of CXCR1 might serve as an adverse prognostic factor for TNM II or III resectable gastric cancer.

### Cox proportional hazards regression analysis shows increased expression of CXCR1 in gastric cancer functions as an independent adverse predictor

Multivariate Cox regression analysis was applied with age, differentiation, lauren classification, TNM stage and CXCR1 expression included. In discovery data set, it turned out that CXCR1 expression, together with TNM stage, was identified as one of the two independent prognosticators for resectable gastric cancer patients’ OS (*P* = 0.001, HR: 2.32, 95% CI: 1.39-3.89 and *P* < 0.001, HR: 2.22, 95% CI: 1.48-3.32, Figure 2D), which was validated in the validation data set (*P* = 0.01, HR: 1.84, 95% CI: 1.16-2.93 and *P* < 0.001, HR: 2.47, 95% CI: 1.65-3.70, Figure 3B). Consequently, our findings suggest that CXCR1 expression could be a reliable independent adverse molecular prognosticator for patients with resectable gastric cancer.

### Prognostic nomogram and comparison of different prognostic models for resectable gastric cancer

**Figure 4:**
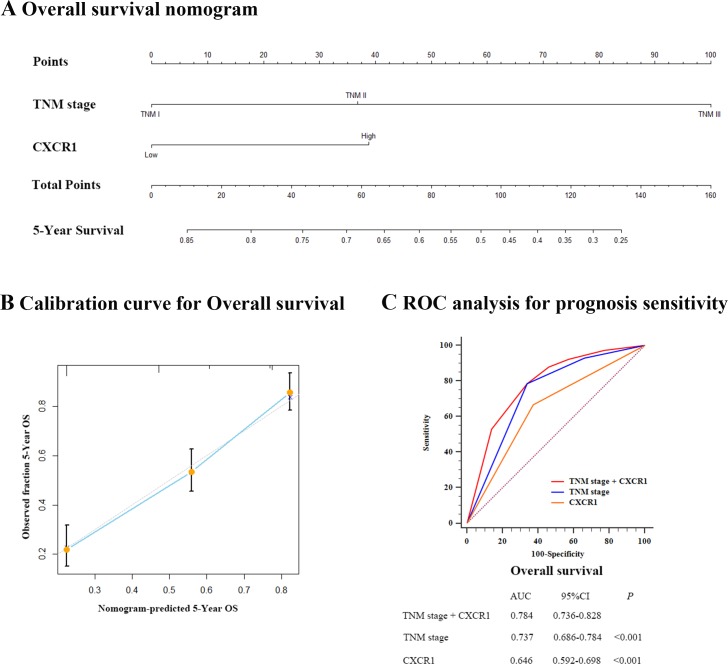
Prognostic nomogram, calibration curve and ROC analysis of prognostic model with CXCR1 expression for resectable gastric cancer (**Panel A**.) Nomogram to predict overall survival (OS) at 5 years after gastrectomy. (**Panel B**.) Calibration curve for nomogram predicted 5-year overall survival performed well with the ideal model. Line of dashes: ideal model; vertical bars, 95% confidence interval. (**Panel C**.) ROC analysis of the prognosis sensitivity and specificity for the overall survival by TNM stage/CXCR1 expression model, TNM stage model, and CXCR1 expression model.

According to the results given by the multivariate analysis, a predictive nomogram predicting OS at 5 years after gastrectomy was constructed in a pooled database to give a quantitative model to stratify CXCR1 patients into different risks (Figure 4A). The predictors, including TNM stage and CXCR1 expression, were both independent prognostic indicators derived from multivariate analysis. Calibration curve for nomogram predicted 5-year overall survival was constructed and performed quite well with the ideal model (Figure 4B).

Next, we aimed to investigate whether the combination of CXCR1 expession with the present TNM stage would improve the predictive accuracy for gastric cancer. According to either Harrell's concordance index (C-index) or Akaike information criterion (AIC), the association of CXCR1 expression with TNM stage was capable of significantly enhancing the prognostic accuracy. As shown in Table 2, the C-index of original TNM staging system and CXCR1 expression alone was 0.6574 and 0.5934 respectively, but improved to 0.6885 when TNM staging system was united with CXCR1 expression. Similary, the AIC of TNM staging system and CXCR1 expression was 1673.93 and 1716.596 respectively, and decreased to 1658.929 once the CXCR1 expression was combined with the current TNM staging system, indicating that the association of CXCR1 expression with the TNM staging system could result in a more reliable and precise prognostic prediction. The result was consistent with ROC prognostic model analysis, which combined two independent prognostic factors, CXCR1 and TNM staging system. Area under the ROC curve (AUC) was applied for the comparison of the prognostic power between the three different models. As a result, the association of CXCR1 expression with the present TNM staging system had more reliable prognostic results than either TNM stage or CXCR1 expression did alone (Figure 4C). The results displayed above demonstrated that the incorporation of CXCR1 into TNM staging system could generate a more precise prognostic model for the prognosis of resectable gastric cancer patients.

**Table 2:** Comparison of the prognostic accuracies of TNM staging system and CXCR1 expression

Model	C-index	AIC
CXCR1	0.5934	1716.596
TNM	0.6574	1673.93
TNM + CXCR1	0.6885	1658.929

Abbreviations: AIC = Akaike information criterion; C-index = Harrell's concordance index; CXCR1= CXC chemokine receptor 1

### CXCR1 expression and benefit from 5-FU based adjuvant chemotherapy (ACT)

**Figure 5:**
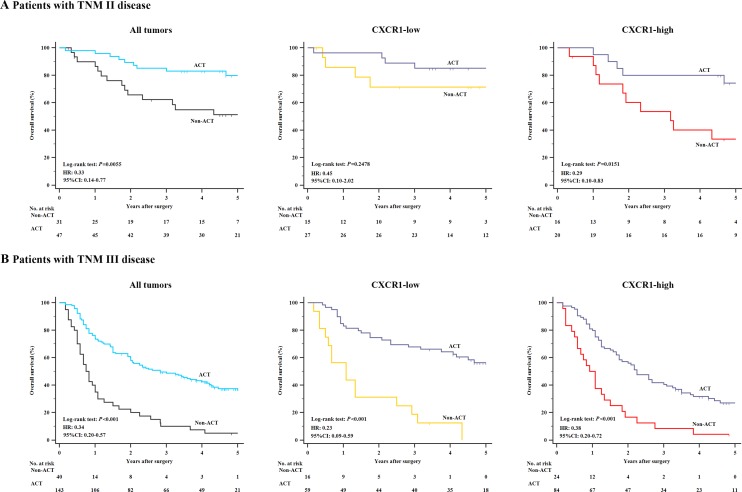
Relationship between CXCR1 expression and benefit from 5-FU based adjuvant chemotherapy (ACT) (**Panel A**.) In TNM stage II disease, CXCR1-high patients (*n* = 36) could significantly benefit from ACT (*P* = 0.0151, HR: 0.29, 95% CI: 0.10-0.83), while no significant difference was observed with respect to overall survival in CXCR1-low subgroup (*n* = 42) when patients were given ACT (*n* = 27) or not (*n* = 15) (*P* = 0.2478). (**Panel B**.) In TNM stage III disease, either CXCR1-low or CXCR1-high patients could benefit from ACT (*P* < 0.001, HR: 0.23, 95% CI: 0.09-0.59 and *P* < 0.001, HR: 0.38, 95% CI: 0.20-0.72).

Ultimately, to assess whether the patients with CXCR1-high tumors could benefit from 5-FU based ACT, we further inspected the relation between CXCR1 expression and overall survival among patients who either received ACT or not. As shown in Figure 5, a preliminary test which involved patients with TNM II and TNM III disease showed a strong correlation between the use of ACT and a higher rate of OS in all tumors. Furthermore, in TNM II disease, CXCR1-high patients could significantly benefit from ACT (*P* = 0.0151, HR: 0.29, 95% CI: 0.10-0.83, Figure 5A), while the use of ACT showed no statistically significant difference in CXCR1-low subgroup with regard to overall survival (*P* = 0.2478, Figure 5A). In TNM III disease, ACT could benefit either CXCR1-low subgroup or CXCR1-high subgroup (*P* < 0.001, HR: 0.23, 95% CI: 0.09-0.59 and *P* < 0.001, HR: 0.38, 95% CI: 0.20-0.72, respectively; Figure 5B). Hence, the results confirmed that treatment with adjuvant chemotherapy could benefit TNM II, CXCR1-high patient population and all TNM III population.

## DISCUSSION

Tracing back to the 19^th^ century, the relation between chronic inflammation and cancer was elucidated by Virchwood for the first time. Anti-inflammatory therapy was reported to be capable of reducing the cancer incidence [28], indicating inflammation might drive several different mechanisms involved in tumor progression and dissemination [29]. As a kind of pro-inflammatory CXC chemokine, IL-8 triggers intracellular downstream signaling pathways through CXCR1 and CXCR2, two G protein-coupled receptors located on cell surface. Elevated expression of IL-8 or CXCR1/CXCR2 has already been featured in cancer cells, tumor-associated macrophages (TAM), and tumor-infiltrating neutrophils (TIN) [19], which suggests that the interaction between IL-8 and CXCR1/CXCR2 may function as a significant regulator under the tumor microenvironment.

Having been researched in a series of cancers, CXCR1 shows a tight correlation with tumor angiogenesis, invasion, metastasis, and drug resistance [30]. In a phase of rapid growth, tumor cells may secrete several chemical signals that trigger tumor angiogenesis and lymphangiogenesis, thus resulting in tumor invasion and metastasis [31]. It was reported that cancer cells would grew to only 1-2mm^3^ in diameter if deprived of blood circulation. When the cancer cells were situated in an area where angiogenesis and blood circulation were possible, however, they could grow far beyond 2mm^3^ [32]. In TNM I gastric cancer, the symptoms are atypical and the tumor progression is not as fast as that in TNM II or III gastric cancer because the disease is still in the early stage. Consequently, CXCR1 expression could not stratify the overall survival of patients with resectable, TNM I gastric cancer as was shown in our findings. The knockdown of CXCR1 was reported to be able to prohibit the proliferation of cancer cells and even induce tumor cell apoptosis in gastric cancer. Also, the knockdown of CXCR1 could lead to the down-regulation of the phosphorylation level of serine/threonine protein kinase (AKT) and extracellular signal-regulated kinase (ERK) 1/2 [33], indicating that AKT and ERK might be involved in the downstream of CXCR1 signal pathway. Another report showed that CXCR1 could up-regulate matrix metalloproteinase-9 (MMP-9) expression by activating JNK/c-Jun and ERK/Ets-1 pathways [34, 35], thus resulting in more aggressive biological characteristics of cancer cells. STAT-3, which is generally considered as an oncogene [36, 37], could also be activated in the downstream of CXCR1 signal pathway [19]. Fortunately, such STAT activation could be significantly inhibited with the use of 5-FU according to a previous breast cancer research [38]. In chronic gastric inflammation, it was likewise suggested that the specific targeting of stomach epithelial STAT3 level might be therapeutically effective in the prevention of gastric carcinogenesis [39].

Patients with TNM II and TNM III gastric cancer were the candidates recruited in our ACT research. It is crucial to identify the patients whose tumor will not only be sensitive to ACT, but lead to more satisfactory overall outcomes so that excessive toxicites could be avoided. In the current study, we therefore inspected the relation between CXCR1 expression and clinical outcomes in ACT-receiving patients. The results suggested that in TNM II patients, those who suffered from CXCR1-high tumors could significantly benefit from ACT. However, the TNM III patients with either CXCR1-high or low tumors could have a longer overall survival. The findings indicated that CXCR1 could be an effective predictor of adjuvant chemotherapy in TNM II patients. And the detection of CXCR1 in TNM II patients could be useful for better selection and management of patients who should be recommended to receive ACT. Nevertheless, the study is retrospective and the number of ACT-receiving patients is relatively small. The results require to be validated in a prospective, larger, multi-centered randomized trial.

In conclusion, our study clarified that CXCR1 expression could predict unfavorable prognosis and be adopted as a novel prognosticator for resectable gastric cancer patients. Combination of CXCR1 expression with present TNM staging system was able to give more precise prognostic information for gastric cancer patients, and might consequently help to identify the patients in need of a much more stringent postoperative follow-up. Furthermore, the findings shed light on individual chemotherapy treatment in gastric cancer patients on the basis of CXCR1 expression, because patients with CXCR1-high tumors tended to have improved outcomes after receiving 5-FU based ACT, especially for the patients with TNM II disease. Thus, the detection of CXCR1 expression in gastric cancer tissues might also assist clinicians to give more suitable clinical treatment and postoperative management strategy to the gastric cancer patients.
